# TGF-β Signaling: A Therapeutic Target to Reinstate Regenerative Plasticity in Vascular Dementia?

**DOI:** 10.14336/AD.2020.0222

**Published:** 2020-07-23

**Authors:** Mahesh Kandasamy, Muthuswamy Anusuyadevi, Kiera M Aigner, Michael S Unger, Kathrin M Kniewallner, Diana M Bessa de Sousa, Barbara Altendorfer, Heike Mrowetz, Ulrich Bogdahn, Ludwig Aigner

**Affiliations:** ^1^Laboratory of Stem Cells and Neuroregeneration, Department of Animal Science, School of Life Sciences, Bharathidasan University, Tiruchirappalli, Tamil Nadu, India.; ^2^Faculty Recharge Programme, University Grants Commission (UGC-FRP), New Delhi, India.; ^3^Molecular Gerontology Group, Department of Biochemistry, School of Life Sciences, Bharathidhasan University, Tiruchirappalli, Tamil Nadu, India.; ^4^Institute of Molecular Regenerative Medicine, Salzburg, Paracelsus Medical University.; ^5^Spinal Cord Injury and Tissue Regeneration Center, Salzburg, Paracelsus Medical University, Salzburg, Austria.; ^6^Velvio GmbH, Regensburg, Germany.; ^7^Austrian Cluster for Tissue Regeneration, Vienna, Austria

**Keywords:** vascular dementia, TGF-β, endothelial cells, pericytes, BBB, hippocampus, neural regeneration, oxidative stress

## Abstract

Vascular dementia (VaD) is the second leading form of memory loss after Alzheimer's disease (AD). Currently, there is no cure available. The etiology, pathophysiology and clinical manifestations of VaD are extremely heterogeneous, but the impaired cerebral blood flow (CBF) represents a common denominator of VaD. The latter might be the result of atherosclerosis, amyloid angiopathy, microbleeding and micro-strokes, together causing blood-brain barrier (BBB) dysfunction and vessel leakage, collectively originating from the consequence of hypertension, one of the main risk factors for VaD. At the histopathological level, VaD displays abnormal vascular remodeling, endothelial cell death, string vessel formation, pericyte responses, fibrosis, astrogliosis, sclerosis, microglia activation, neuroinflammation, demyelination, white matter lesions, deprivation of synapses and neuronal loss. The transforming growth factor (TGF) β has been identified as one of the key molecular factors involved in the aforementioned various pathological aspects. Thus, targeting TGF-β signaling in the brain might be a promising therapeutic strategy to mitigate vascular pathology and improve cognitive functions in patients with VaD. This review revisits the recent understanding of the role of TGF-β in VaD and associated pathological hallmarks. It further explores the potential to modulate certain aspects of VaD pathology by targeting TGF-β signaling.

## Epidemiology of Vascular Dementia

Dementia describes a set of clinical symptoms affecting cognitive, motor and behavioral aspects, particularly in the geriatric population [1, 2]. Approximately 50 million people suffer from dementia, and its incidence doubles every two decades creating a huge socioeconomic and health care burden worldwide. Vascular Dementia (VaD) contributes to 20-40% of all dementia cases making it the second most common form of dementia after Alzheimer’s disease (AD) [3]. The occurrence of VaD varies among populations. For example, unlike AD, the prevalence of VaD is higher in the Asian compared to the Caucasian population [4]. However, VaD frequently coexists with AD and it is often difficult to distinguish the symptoms between VaD and AD. While AD poses a strong gender bias towards women, the prevalence of VaD is higher in men most likely due to cerebrovascular risk factor being more prevalent in males [5]. Thus far, there are no curative, reparative or regenerative treatments available and therefore, development of therapy for VaD has become an essential but unmet need.

## Risk factors of Vascular Dementia

**Figure 1.**
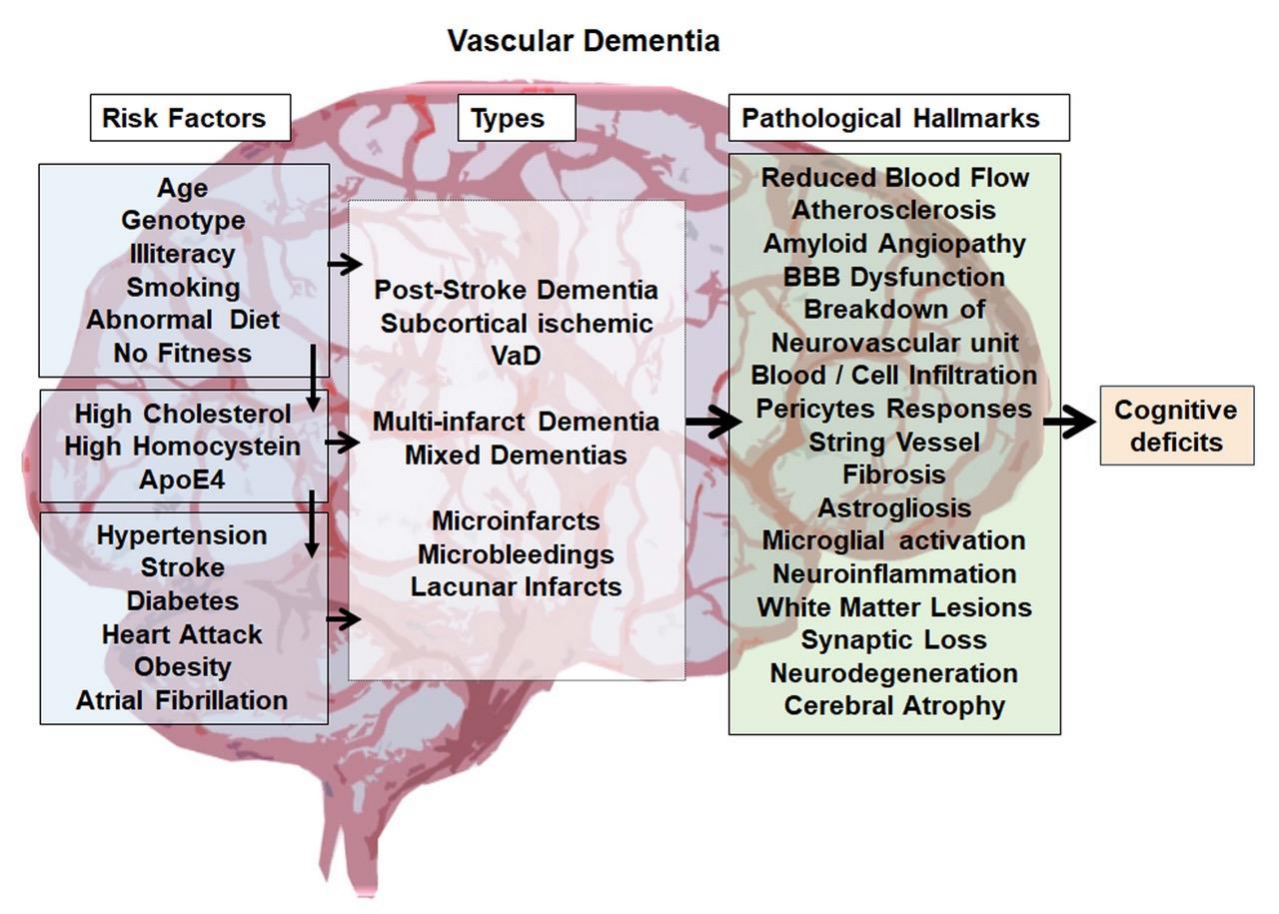
**An overview of Vascular Dementia**. The figure summarizes the possible biological and lifestyle risk factors, various forms of VaD, pathological hallmarks of VaD and indicates cognitive deficit as the final outcome of the disease.

Risk factors for VaD are multifactorial and include aging, illiteracy, genetic predisposition, abnormal conditions and diseases such as hypertension, stroke, diabetes, obesity, coronary infarction, atrial fibrillation, and atypical biochemical blood parameters such as high cholesterol and homocysteine levels. Various lifestyle factors such as smoking, unhealthy diet and physical inactivity have been associated with an increased risk of VaD [2, 6] (Fig. 1). In consequence, reducing or avoiding such risk factors, if possible, is strongly recommended for the prevention of VaD. These include maintenance of healthy blood pressure, prevention or control of diabetes, smoking cessation, maintaining physical fitness and controlling blood cholesterol levels. The rapid increase in cerebrovascular diseases including stroke worldwide suggests that the currently used primary preventive actions against stroke and cardiovascular diseases have very limited efficacy, most likely due to the fact that they function on a voluntary basis with limited self-motivation [7]. This leaves a huge challenge to the scientific community to develop effective therapies to successfully treat VaD.

## Classification of Vascular Dementia

Based on the unstructured diagnostics criteria, VaD has been classified into different types. In 1997, Konno S et al, proposed eight possible forms of VaD, 1) multi-infarct dementia, 2) post-stroke strategic infarcts dementia, 3) multiple subcortical lacunar lesions, 4) Binswanger's disease or arteriosclerotic subcortical leukoence-phalopathy, 5) mixed type of VaD co-occurring with types 1, 2 and 3, 6) haemorrhagic lesion-induced dementia, 7) subcortical vascular dementia, and 8) mixed type of VaD with AD [8, 9]. The aforementioned sub-types of dementias based on vascular dysfunction include several neuropathological syndromes which are all characterized by a reduced cerebral blood flow (CBF) leading to cognitive dysfunctions and memory loss [2]. Some dementias occur after major and/or recurrent strokes in strategically important brain areas and are often considered a major stroke sequel based on large vessel pathology rather than an independent disease [10, 11]. Less common forms of VaD are various types of vasculitis and inherited diseases that affect vessel integrity, such as CADASIL, or the three-prime repair exonuclease (TREX)-1-related thrombotic microvascular disease [12-15]. The predominant form of VaD is based on cerebral small vessel disease, leading to vascular pathologies in deep penetrating arteries and resulting in numerous minor ischemic-hypoxic lesions (multiple lacunes) in deep cortical areas or subcortical areas [16]. Irrespective of the specific subtype, risks include atherosclerosis of cerebral arteries and cerebral amyloid angiopathy [2, 6] (Fig. 1).

## Clinical Presentation of Vascular Dementia

In the early phase, VaD can be hardly differentiated from other cognitive deficits such as mild cognitive impairment [6, 17]. As VaD advances, depending on the brain areas that are affected, clinical manifestations of severe cognitive deficits, loss of memory and motor disorders such as gait instability, psychiatric symptoms such as aggressiveness and depression, loss of bladder function, and increasing difficulties with daily routines become clearly evident [1, 2, 6, 17, 18]. Overlapping symptoms of VaD with other dementias and its occurrence with mixed pathologies creates a huge challenge for accurate diagnosis [17, 19]. Besides the obvious clinical symptoms, diagnosis of VaD requires the proof of impaired cerebral blood flow in the brain and vascular imaging such as MRI, CT, angiography and carotid ultrasound [8, 20-23].

## Pathological hallmarks of Vascular Dementia

Irrespective of its origin, the pathogenesis of VaD is quite multifaceted. Upon a closer view, this can involve atherosclerosis, amyloid angiopathy, microbleeding and micro-stroke, blood-brain-barrier (BBB) dysfunction and leakage, infiltration and invasion of blood-derived factors such as circulating immune cells, extracellular vesicles and molecules, abnormal vascular remodeling, endothelial cell death and string vessel formation, pericyte responses, fibrosis, astrogliosis, microglia activation, oxidative stress, neuroinflammation, demyelination and white matter lesions, deprivation of synapses and neuronal loss (Fig. 1) [6, 17-19]. Spontaneous intracerebral hemorrhage can be one of the early pathological features of VaD [24-26]. In the lesioned or degenerative brain, BBB disruption leads to an influx of blood molecules, invasion of blood cells and microbial pathogens into the brain and is associated with inflammatory and immune responses, which can initiate multiple pathways of neurodegeneration [27]. The pathogenic mechanisms rendered by BBB breakdown leads to neuronal injury, synaptic dysfunction, loss of neuronal connectivity and neuronal loss, which have been described in neurodegenerative disorders including VaD [28-31].

The complex pathophysiology in VaD affects neuronal networks involved in behavior, intellectual process, execution and memory, ultimately leading to cognitive deficits [6, 9] (Fig. 1). Considering and accepting this complexity imposes a huge challenge in drug and therapy development for VaD. Most likely, the classical view of a drug with “single mode of action” is inappropriate for a disease like VaD. In consequence, any kind of therapeutic strategy for the diseased brain, including VaD, needs to address a plethora of pathological aspects in a multimodal fashion and needs collectively to include neuroprotection, plasticity, as well as regeneration in the brain. Such an approach most likely needs to be centered around the vascular niche and neurovascular unit, where the various affected cell types meet [32, 33].

## Molecular Targets of Vascular Dementia: TGF-β as a potential candidate

On the molecular and genetic level, a few candidates for pathogenesis in VaD have been discussed. For example, polymorphisms and differential expression analyses have identified apolipoprotein E (APOE), methylene-tetra hydrofolate reductase (MTHFR), paraoxonase 1 (PON1), tumor necrosis factor (TNF)-alpha, vascular endothelial growth factor (VEGF), and transforming growth factor (TGF)-β1 as potential candidate factors involved in the pathogenesis of VaD [30, 34]. Among them, TGF-β1 has gained a remarkable scientific interest as it plays a crucial role in angiogenesis, neuroprotection, neuroimmune functions, neural regeneration and synaptic plasticity, which are responsible for cognitive functions in the physiological state [35-38].

**Figure 2.**
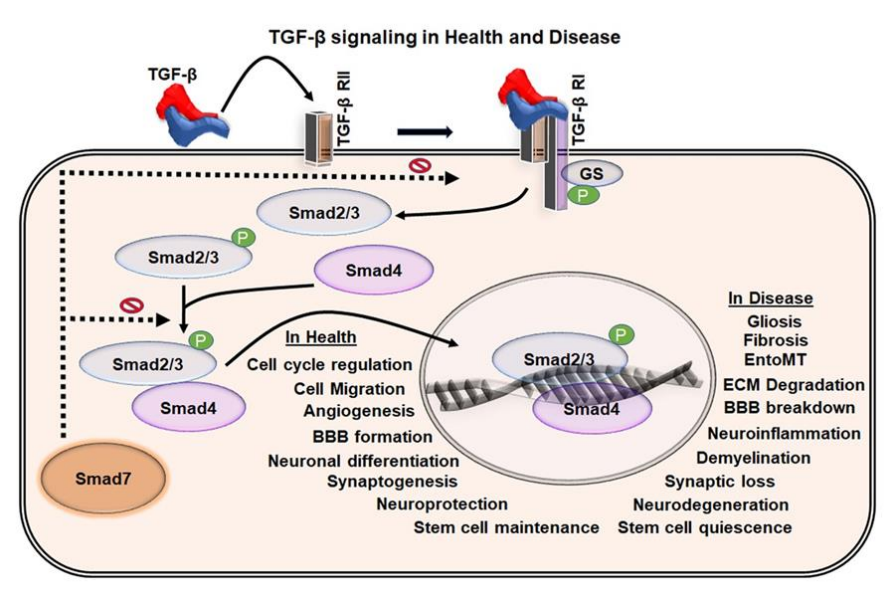
**TGF-β signaling in health and disease**. Schematic representation of the TGF-β mediated Smad signaling through the TGF-β receptors. TGF-β binds to TGF-β receptor II, which induces the activation of TGF-β receptor I and activates the phosphorylation of Smad2/3 in the cytoplasm. The activated Smad2/3 binds to Smad4 in the cytoplasm and mobilize into the nucleus where they act as a transcription factor. This leads to various cell-type dependent responses essential for the tissue homoeostasis and various physiological functions. Dysregulation of the TGF-β pathway leads to abnormal cellular events and pathological hallmarks that are all part of the VaD pathology.

TGF-β proteins (TGF-β 1, 2, and 3) are secreted in an inactive form by various cells in various tissues and organs [35]. Thrombospondin (TSP)-1, plasmin, and matrix metalloproteinase (MMP)-2 and -9 trigger the activation process of TGF-β [39]. Besides, the generation of reactive oxygen species (ROS) and the accumulation of an acid milieu in damaged tissues facilitate the activation of TGF-β, thereby leading to the release of TGF-β ligands for the receptor-mediated activation of Smads [40-43]. In particular, TGF-β induces the phosphorylation of Smad2 and Smad3 that bind to Smad4 in the cytoplasm and translocates it into the nucleus [35, 38, 44]. This heteromeric complex acts as a transcription factor to control the expression of various target genes. Thus, TGF-β facilitates transcriptional regulation to create a complex and synergistic higher-order signaling network in various complex biological mechanisms [35, 45] (Fig. 2).

TGF-β1 regulates cell proliferation, differentiation, maturation and survival of various neuronal and non-neuronal cell types in the brain [38, 46]. Also, it regulates angiogenesis, BBB integrity, neuroinflammation, neuroplasticity and neuronal function [35, 38, 47-50]. The choroid plexus, as well as astrocytes, microglia, and endothelial cells, have been the major sources of TGF-β1 in the brain [51-53]. In the brain, TGF-β1 is typically elevated after acute brain injury and in chronic neurodegenerative pathologies, where it is the main driver of the astroglial scar formation, fibrosis and sclerosis [54-57].

Elevated levels of TGF-β1 in response to cerebro-vascular pathologies, neurodegeneration, neuro-inflammation, and neuroregenerative failure appear to be involved in the progression of dementia [34, 58-62]. Also, genetic variations in TGF-β and TGF-β receptors are considered as the prominent risk factors for VaD. Thus, TGF-β signaling is a therapeutic target candidate in various forms of cognitive disorders. Detailed information on the possible involvement of TGF-β in VaD is limited. In the present review, we focus on TGF-β and its potential role in VaD, in particular, due to its multimodal effects in the brain and neuropathogenesis.

## TGF-β and pathogenesis of VaD

Evidence accumulating from human genetic association studies, from expression analyses and animal experiments strongly suggest that TGF-β plays a role in the pathogenesis of VaD. For example, a meta-analysis of 69 studies with 4,462 cases and 11,583 controls identified polymorphisms in the TGF-β1 gene to be associated with a higher risk for VaD, microinfarcts, ischemic stroke and cerebral amyloid angiopathy [34, 63, 64]. Also, TGF-β1 levels are increased in monocyte-derived macrophages, plasma, in the cerebrovascular system, as well as in the cerebrospinal fluid in patients and animal models of VaD [60, 62, 65-69]. and in hypertensive, diabetic and ischemic stroke patients [70, 71]. In CARASIL, a hereditary small vessel disease, mutations in the serine protease HTRA1 gene result in increased TGF-β signaling, provoking multiple effects including vascular fibrosis and extracellular matrix synthesis [72, 73].

Strong evidence for the role of TGF-β1 in VaD is derived from studies using mice overexpressing TGF-β1. These mice have originally been generated by Tony Wyss-Coray and colleagues and are characterized by massive deposition of ECM and the formation of hydrocephalus [74]. This mouse model has a higher susceptibility to neuroinflammation triggered by experimental autoimmune encephalitis [75], accelerated disease progression in the context of amyotrophic lateral sclerosis (ALS) [76], severe formation of amyloid plaque pathology in the context of an AD background [69], microvascular degenerations [62], reduced level of neurogenesis [77], and most importantly, learning deficits [78]. More specifically in the context of VaD, these mice show reduced functional vascular reactivity in the sense of a lower dilatation as well as contraction behavior in response to stimuli, capillary degenerations resulting in the formation of string vessels, vascular fibrosis and calcification [79]. Importantly, in the scenario of VaD, the TGF-β1 overexpressing mice show reduced CBF, which correlates with the deposition of amyloid plaques in the blood vessels [80].

## Cerebral blood flow and TGF-β in VaD

Reduced CBF is the hallmark of VaD, which may be the result of very diverse conditions such as atherosclerosis, cerebral amyloid angiopathy, microbleeding, micro-strokes and larger territorial stroke. Such damages typically induce a rise in TGF-β1 levels, which locally affects neurodegenerative and neuroregenerative events. The causative role of TGF-β in priming and development of CBF and VaD is obscure. Nevertheless, in patients with hypertension, the main risk factor for VaD, plasma levels of TGF-β1 are markedly increased [81] and this correlates with hypertension-induced tissue and organ damage such as nephrosclerosis and kidney diseases [82]. Thus, the observed changes in CBF seen in neurovascular pathologies might be strongly associated with the increased levels of TGF-mediated defects in the cellular components of the BBB.

## The roles of TGF-β in BBB functions

The BBB serves as a remarkable physical and biochemical barricade between the brain and the blood. The BBB is primarily built up by endothelial cells lining the microvessels and sealed through tight junctions. The vessel wall is ensheathed by pericytes and surrounded by end-feet of perivascular astrocytes, microglial cells, and neurons, called neurovascular unit (NVU) [83-87]. In the healthy brain, the BBB protects neurons from blood-derived factors and maintains levels of neurochemical parameters of the internal brain milieu, which are essential for neuroprotection, neuronal functioning and synaptic transmission [84, 88]. This specialized function of the BBB is maintained by the establishment of a mutual trophic interference and three-dimensional vicinity between endothelial cells, pericytes and astrocytes in the brain.

**Figure 3.**
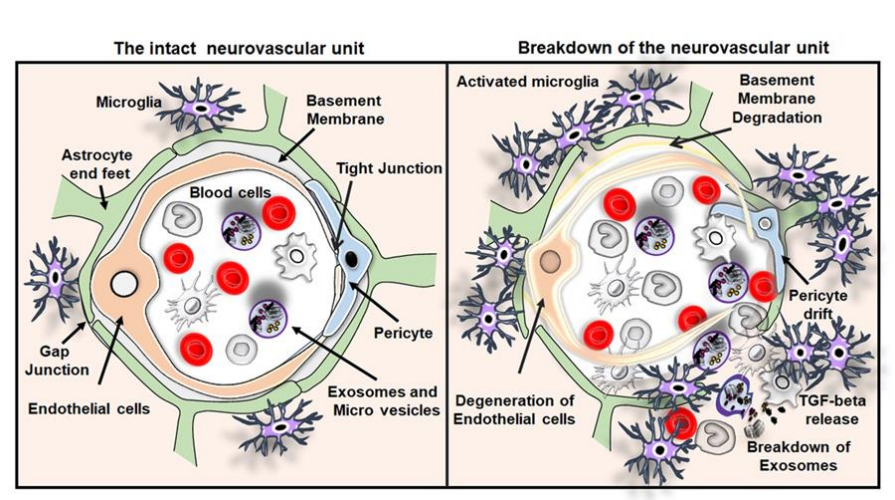
**The intact neurovascular unit and breakdown of the neurovascular unit in VaD**. Illustrations of the intact neurovascular unit that typically contains normal blood cells, endothelial cells, basement membrane ensheathed by pericytes and end feet of astrocytes in the brain. The figure also represents the breakdown of the neurovascular unit resulting from the BBB disruption, the crucial pathological hallmarks of VaD such as endothelial cell death and drift of pericyte. The activation of immune cells interaction between the activated microglia and peripheral blood cells leading to the release of TGF-β and breakdown of exosomes and release of macrovesicles in the brain of subjects with VaD.

The potential relationship between TGF-β and the BBB is demonstrated in gene knockout studies as complete deletion of TGF-β1, TGF-βR1, TGF-βR2, TGF-βR3 (endoglin) and of Smad4. Those resulted in defects in blood vessel formation and embryonic lethality due to an improper attachment between endothelial and mesothelial cells. Systemic inhibition of TGF-β by soluble endoglin in pregnant rats contributes to the development of Preeclampsia, a disease condition associated with increased BBB leakage in pregnant women. Moreover, a Cre-LoxP based conditional deletion of Smad-4 specifically in brain endothelial cells resulted in BBB breakdown due to disruption in the interaction between pericytes and endothelial cells. Besides, TGF-β contributes to the construction of basement membrane through the secretion of ECM components in endothelial cells and pericytes that are integral parts of the BBB [89-91]. Moreover, the increased autocrine TGF-β signaling in pericytes in diabetic encephalopathy induces basement membrane hypertrophy disrupting the BBB [92]. Taken together, TGF-β plays a key role in the recruitment of pericytes and maintenance of the contact between endothelial cells and pericytes at the BBB (Fig. 3). However, the TGF-β mediated recruitment of astrocytes to the BBB and the complete molecular basis of interactions among cellular components of the BBB remain largely unknown.

In the context of cancer, increased TGF-β facilitates metastasis of tumor cells through the down-regulation of junctional adhesion and tight junction proteins upon the induction of matrix metalloproteases (MMPs) [35, 93]. Similarly, in neuroinflammatory conditions, down-regulation of the expression of junctional adhesion and tight junction proteins in the blood vessel and increased expression of MMPs in association with abnormal TGF-β signaling primes the leakage of BBB, which in turn leads to degeneration of endothelial cells, pericytes drifting and string vessels in the neurovascular systems [94]. Based on the observation from the ablation of the TGF-β signaling pathway in pericytes through retroviral-mediated expression of a truncated C-terminal intracellular kinase domain of TGF-βR2, Sieczkiewicz GJ et al. have described that endothelial cell-specific secretion of TGF-β inhibits the mitotic activities of pericytes through the activation of pericyte contractile protein leading to the BBB breakdown [95].

## Abnormal levels of TGF-β and BBB dysfunction in VaD

In a preclinical genetic mouse model of VaD with a point mutation in the NOTCH3 gene, TGF-β signaling is disrupted, and pericyte numbers are reduced leading to BBB breakdown. Notably, endothelial-specific Smad4 deficient animals had increased BBB permeability, BBB breakdown and intracranial haemorrhages [96]. Moreover, the animal models with endothelial cell-specific depletion of TGF-receptors including endoglin have cerebrovascular defects and BBB dysfunctions [97-101]. In humans, mutations in the TGF-β receptors have been linked to hereditary haemorrhagic telangiectasia (HHT). HHT is an autosomal dominant vascular dysplasia, caused by mutations in endoglin and ALK1, which is characterized by dilated vessels and arteriovenous malformations [102]. Eventually, the transgenic model of HHT is characterized by significant structural alterations in cerebral blood vessels, pericyte drift and BBB breakdown [103]. However, the role of TGF-β in the BBB breakdown and vessel leakage in VaD is currently not clear. While many cerebrovascular pathologies, including VaD, have been characterized by the increased level of TGF-β, defects in its downstream pathways and impact of the crosstalk between abnormal levels of TGF-β and other signaling mechanism needs to be considered. Future studies directed towards the potential involvement of TGF-β in BBB dysfunction would signify the therapeutic options for VaD.

## The role of TGF-β in vascular remodeling in VaD

Vascular remodeling (VR) is a dynamic process of structural plasticity of blood vessels in which the blood vessel cells are constantly altered at the level of cell growth, cell migration, cell death, and at the level of production and degradation of ECM components present in the basement membrane [104-108]. This can be in response to variation in blood flow and hypertension, and also as a consequence of lesions [107, 109, 110]. Interactions between vascular cells mediate the regulation of VR through signaling of growth factors, vasoactive substances and hemodynamic stimuli [109, 111]. Endothelial cells contribute to vascular hemostasis, interact with circulating blood cells, provide resistance over the hemodynamic tension and mechanical stimuli from blood pressure, and respond to inflammation [112-114]. TGF-β signaling is one of the key pathways involved in the regulation of VR [115, 116]. In general, mechanical stimuli are crucial regulators of growth as well as degeneration of endothelial cells that contribute to VR [117, 118]. Spontaneously hypertensive rats with altered hemodynamics exhibited increased levels of endothelial heparan sulfate proteoglycan (HSPG), another key component of basement membrane regulated by TGF-β-Smad signaling [119]. While TGF-β1 deficient mice are embryonic lethal due to defects in vasculogenesis [120], in vivo induction of TGF-β1 appears to mediate angiogenesis. TGF-β has been reported to exert differential effects on vascular endothelial cells. In general, the formation of new capillaries from preexisting blood vessels is mediated by TGF-β1 induced expression of endothelial cell VEGF through fibroblast growth factor (FGF-2)[47]. Recently, Schlecht et al. described that TGF-β is required for physiological vasculogenesis in the eye, as deletion of TGF-βR2 especially in endothelial cells of the eye results in choroidal neovascularization [121]. Moreover, in vitro experiments of cultured endothelial cells suggest that TGF-β inhibits the mitotic activity and induces cell death of endothelial cells [122, 123]. In addition, prolonged treatment of vascular endothelial cells together with TGF-β and FGF-2 abolishes the mitogenic effect of FGF-2 on endothelial cell function and migration through the deactivation of plasminogen activator [124, 125]. Apparently, there exists a positive feedback loop from TGF-β1 induced VEGF expression that in turn boosts the expression of TGF-β, which results in the activation of p38 mitogen-activated protein kinase (MAPK) leading to the degeneration of endothelial cells during chronic neuroinflammation and vascular pathogenesis [126]. When endothelial cell death occurs, the basement membrane persists as empty tubes known as string or ghost vessels. Such TGF-β induced degeneration in endothelial cells appears to induce string vessels in the brain [126], which are often associated with neurovascular pathogenesis in AD and VaD [59]. Microvessels isolated from brains of AD subjects with vascular pathologies are also reported to release TGF-β [66]. In the context of cerebral amyloid angiopathy, TGF-β appears to induce the generation of amyloid-β depositions in blood vessels. Thus, TGF-β mediated accumulation of amyloid-β might also be closely associated with the endothelial cell death leading to cerebrovascular pathologies in VaD [127, 128].

Recently, endothelial to mesenchymal transition (EndoMT) has been proposed as a potential mechanism involved in VR and BBB breakdown in cerebrovascular pathologies [129, 130]. EndoMT is a cellular process by which endothelial cells dedifferentiate into mesenchymal cells leading to degradation of vascular basement membrane and disintegration of endothelial cells [131]. Moreover, physiological properties and functions of endothelial cells are impaired, and they acquire a migratory phenotype. Notably, cardiac fibrosis, metastatic conditions, systemic sclerosis, multiple sclerosis, stroke, cerebral cavernous malformation, vein stenosis and diabetic retinopathy have been characterized by EndoMT [132-134]. Troletti et al. demonstrated that neuro-inflammation-mediated TGF-β and IL-1 β play a key role in EndoMT in the vasculature of human post-mortem brains with multiple sclerosis and cerebrovascular abnormalities [129]. Moreover, inhibition of TGF-β signaling by neutralizing antibodies, endothelial cell-specific Smad2/3 deletion, shRNA-mediated knockdown of Smad2 and haploinsufficiency of Smad3 minimize EndoMT [135, 136].Therefore, abnormal circulation of TGF-β might contribute to VR and breakdown of BBB through EndoMT in VaD.

## Pericyte responses in VaD and the role of TGF-β

Pericytes are multifunctional cells which ensheath the blood vessel and contribute to the regulation of capillary diameter, vasoconstriction and vasodilation, capillary blood flow and extracellular matrix protein secretion [33, 137, 138]. Moreover, pericytes have an immune-modulatory role in the brain [139]. With reference to the origin of the pericytes, both the mesenchymal and neural crest cells have been proposed to give rise to pericytes in many organs. It has recently been reported that pericytes play important roles in the homeostasis of the brain [140-142]. Pericytes are positioned within the BBB between endothelial cells, astrocytes and neurons [138]. Thus, pericytes can integrate and process signals from their adjacent cells to mediate diverse functional responses that are important for brain function in health and disease, including the regulation of the BBB permeability, angiogenesis, clearance of toxic metabolites, phagocytotic activity, capillary hemodynamic responses and neuroinflammation.

In human post-mortem AD brains and transgenic animal models of AD, pericytes are dysfunctional and they undergo degeneration [143-145]. Also, levels of soluble platelet-derived growth factor receptor β (PDGFR-β), a biomarker for pericyte death, are increased in cerebrospinal fluid (CSF) of subjects with mild dementia and in transgenic AD animals [138, 146, 147]. Thus, there is evidence for pericytes being involved in VaD, although the underlying mechanisms are largely unknown. Nevertheless, a familial form of VaD, CADASIL is associated with dysfunction of pericytes in the brain [148].

Under physiological conditions, TGF-β regulates the proliferation and differentiation of pericytes and their attachment to endothelial cells to facilitate CBF and angiogenesis [33, 137, 149, 150]. Apparently, the interaction between endothelial cells and pericytes leads to the expression of TGF-β by both cell types, with differential responses to the TGF-β activation [151-153]. In fibrosis, hypoxia, brain injury, brain metastasis, neurodegenerative conditions and cerebrovascular pathologies, microglia and astrocytes are activated and produce large amounts of TGF-β1 [70, 74, 154, 155]. Elevated levels of TGF-β in the brain stimulate the proliferation of pericytes and trans-differentiation of pericytes into myofibroblasts [156]. Controversial to that, Rustenhoven et al. have recently described that overexpression of TGF-β suppresses the proliferation of pericytes and reduced the phagocytic ability of pericytes [150]. Moreover, a genetic model of pericyte depletion shows BBB disruption-mediated neuro-degeneration [157, 158]. Besides, a transgenic APOE4 mouse model has signs of BBB abnormality and neurodegeneration associated with dysfunctions of brain pericytes [159]. The aforementioned two genetic models have been shown to exhibit cognitive dysfunctions in association with abnormal TGF-β signaling which might be speculated to result from VaD. Thus, TGF-β mediated pericyte responses in neurocognitive disorders including VaD require detailed scientific investigations.

## Role of TGF-β on exosomes and microvesicles in VaD

Many physiological and pathophysiological processes are regulated by extracellular vesicles, which are involved in short and large distance intercellular communications independent of direct cell-to-cell contact [160, 161]. Extracellular vesicles contain high amounts of miRNAs and proteins originated in many different cells including peripheral endothelial cells. At present, exosomes and microvesicles have gained huge attention in neurodegenerative diseases including dementia, for example in AD [162-165]. Of note, in the context of AD and VaD, the intermediate products of amyloid-β, such as lower molecular weight amyloid-β oligomers and protofibrils, are transported in extracellular vesicles spreading from neuron to neuron. Those are neurotoxic and act as a source for further aggregation of amyloid-β in the brain [165]. Interestingly, intracellular amyloid-β is often found to be co-localized with exosomes [166, 167]. Remarkably, mobilization of exosomes is regulated by TGF-β. For example, TGF-β is involved in the transport of MMP2 containing exosomes in malignancy disorders [168]. Therefore, it can be speculated that the increased levels of TGF-β in brains with AD and VaD may play a key role in transport and accumulation of amyloid-β responsible for neurodegeneration.

## Neuroinflammation and astrogliosis in VaD and the role of TGF-β

Neuroinflammation, like in many other neuro-degenerative diseases, is one of the crucial hallmarks of VaD [146]. It describes a wide range of inflammatory responses within the CNS and is mediated by the production of cytokines, chemokines, and reactive oxygen species. At the cellular level, neuroinflammation involves mainly myeloid cells, i.e. microglia, monocytes, and macrophages, and other peripherally derived immune cells infiltrating the CNS, as well as astrocytes. In response to vascular injuries, microglia are the first cells to respond. Initially classified as M1 pro-inflammatory and anti-regenerative, and M2 anti-inflammatory and pro-regenerative, it is now clear that microglia appear in many different subtypes and phenotypes, with high plasticity and transition between the various types [169-171]. Though microglia have altered activity in VaD even before clinical symptoms and neuropathological hallmarks arise [85, 172], their precise role in VaD is unclear. Nevertheless, under inflammatory conditions, microglia are producers of IL-1b [173], and higher expression of IL-1b has been reported in the brain of hypertensive rats [174]. Also, microglia may contribute to CBF alterations and hypertensive vascular pathology in the brain [175]. Besides microglia, perivascular macrophages might be involved in particular. Monocytes are attracted to the vascular wall by vascular damage, where they differentiate into macrophages, locally secrete matrix proteins, vasculo-protective and pro-angiogenic factors as well as several other growth factors [176], promoting angiogenesis and functional recovery [177].

**Figure 4.**
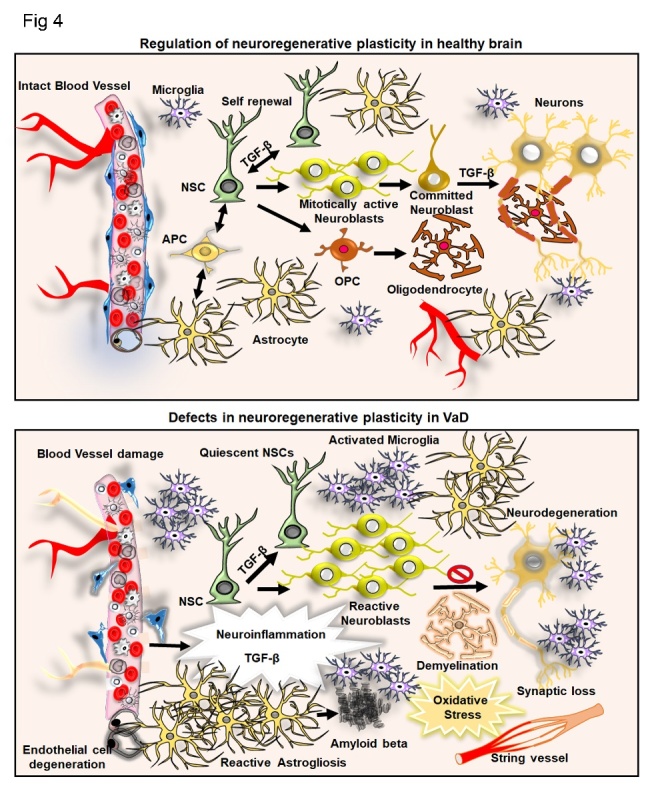
**TGF-β and its involvement in neuroinflammation and neurogenesis in the control brain in the context of VaD**. Illustration of the regulation of adult neurogenesis by TGF-β at the level of neural stem cell (NSC) self-renewal, transformation of neuroblasts and neuronal differentiation. The intact blood vessel, astrogenesis through astrocyte precursor cell (APS) and oligodendrogenesis from oligodendrocyte precursor cells (OPC) and resident microglial cells are indicated. In the parallel side, the figure indicates the aberrant regulation of adult neurogenesis, activated astrogliosis, microglial activation, accumulation of amyloid β, formation of string vessel, demyelination, neurodegeneration resulting from neuroinflammation mediated abnormal levels of TGF- β in VaD.

Neuroinflammation, in particular, microglia activity is strongly regulated by TGF-β. It seems that TGF-β signaling maintains microglia homeostasis. For example, conditional loss of TGF-β signaling in microglia resulted in upregulation of activation and priming markers indicative of a pro-inflammatory stage [178]. Also, TGF-β1 modulates the microglial phenotype and promotes recovery after intracerebral hemorrhage [179]. In the context of VaD, TGF-β regulates microglial activity and induces resistance to hypertension. For example, removal of TGF-β or blocking its signaling before hypertension induction accelerated hypertension progression, and supplementation of TGF-β1 substantially suppressed neuroinflammation and generated immunosuppressive microglia [180]. In summary, TGF-β has, in the context of neuroinflammation and microglia, anti-inflammatory and homeostatic functions (Fig. 4).

Besides monocytes and macrophages, astrocytes are involved in the pathology of VaD. For example, astrogliosis is an acute (7 days) and chronic (3 months) response to artery occlusion in rats [181-183]. Here, in contrast to the effects on microglia, TGF-β seems to have detrimental effects. For example, TGF-β induces astrogliosis and is the main driver of gliotic scarring [74]. In the context of VaD, TGF-β overexpression induces a thickened vascular wall due to accumulation of extracellular matrix proteins [184]. In summary, TGF-β plays a crucial role in neuroinflammation, in particular, in the context of microglial and astroglial cells.

## Demyelination and white matter lesions in VaD and the role of TGF-β

White matter lesions are defined by white matter hyperintensities in magnetic resonance imaging and are a hallmark of VaD. At the cellular level, white matter lesions are characterized by myelin and oligodendrocyte loss, by axonal damages associated with axonal thinning and the accumulation of varicosities, and by loosening of the axon-oligodendrocyte adhesion [185-188]. Therefore, myelin protection and remyelination are clear targets in VaD. This is, in particular, the case in the aged brain, where remyelination is insufficient [189, 190].

The role of TGF-β in de- and remyelination is still controversially discussed. For example, TGF-β1 is associated with the progression of intracranial deep white matter lesions in humans [191]. In contrast, TGF-β1, which is present in higher levels in the systemic environment, promotes oligodendrocyte maturation, while lower levels in circulating TGF-β prevent remyelination in the spinal cord after toxin-induced demyelination [192]. Also, TGF-β promoted subcortical white matter remyelination, while TGF-β receptor deletion in oligodendroglial progenitors prevents their development into myelinating oligodendrocytes [193].

## Loss of synapses and neurodegeneration in VaD and the role of TGF-β

Synaptic loss and neurodegeneration are the foremost neuropathological hallmarks that reflect various forms of dementia [194-196]. With reference to synaptic function, a wide range of proteins involved in vesicular trafficking, synapse structure and signal transduction are known. Synaptophysin, a presynaptic vesicle-specific protein is considered as a marker of synaptic integrity [197-199]. Protein expression studies on synaptic membrane preparations from various brain regions of subjects with AD and VaD revealed a reduction of synaptophysin levels in the hippocampus [200]. Postsynaptic density protein 95 (PSD-95) is a membrane-associated guanylate cyclase family of proteins located in dendritic spines and is required for synaptic plasticity [201, 202]. The expression of PSD-95 is reduced significantly in AD, mild cognitive impairment and VaD [203, 204]. Synaptosomal-associated protein 25 (SNAP-25) is part of the SNARE complex involved in synaptic vesicle membrane docking and fusion, and is present in the presynaptic terminal participating in exocytosis and neurotransmitter release [205, 206]. A significant reduction in the expression of SNAP-25 is reported in AD brains, in frontotemporal dementia and VaD [199, 203, 207]. Drebrin (developmentally regulated brain protein), an F-actin binding protein involved in dendritic spine morphogenesis, is also drastically reduced in AD and mild cognitive impairment but increased in VaD [17, 203, 208]. The rationality of the increased level of drebrin in VaD is not clear but it might be a compensatory response to the ischemia related pathomechanisms caused by VaD [203]. The dysregulation in the expression of presynaptic and synaptic proteins that are involved in synaptic function are correlated with a significant loss of synapses in the brains of subjects with different forms of dementia.

A role of TGF-β in synaptic protein expression is not known, but TGF-β certainly modulates spines and spine densities. For example, the brain-specific knock down of TGF-β1 in mice showed a reduction in the dendritic spine density and a defect in neurotransmission in the hippocampus [209]. Treatment of the sea-snail Aplysia with TGF-β1 promotes sensorimotor synapses and reduces synaptic dysfunctions through the activation of MAPK and BDNF-mediated CREB phosphorylation [210, 211]. Also, deficiencies in the expression of TGF-β, deletion of TGF-β receptors and pharmacological inhibition of Smad2/3 phosphorylation induces neurodegeneration and synaptic dysfunctions [54, 178, 212-215]. Astroglial-specific production of TGF-β in aged transgenic mice expressing the amyloid beta precursor protein (APP) minimizes dystrophic neurites and rescues neurodegeneration [51, 216]. However, prolonged activation of microglia and thereby induced presence of an increased amount of TGF-β in the brain is linked to the synaptic loss in AD and VaD [155, 217-220]. Also of relevance, the clinical signature of various forms of dementia is most likely linked to the synaptic dysfunctions rather than neurodegeneration [221, 222].

## The regulation of adult hippocampal neurogenesis by TGF-β signaling

Defects in hippocampal neurogenesis are proposed to contribute to cognitive deficits in dementia [223]. Recently, we provided experimental evidence for the widespread expression of the TGF-β signaling components including Smad in the intact adult brain [38]. Immunoblotting and immunocytochemistry analyses revealed that considerable expression of TGF-βR2 is evident in the olfactory bulb cortex, striatum and cerebellum of the adult rat brain. Expression of the TGF-βR2 is relatively low in the neurogenic niches such as the hippocampus and the SVZ. However, we have noticed high expression levels of TGF-βR1 as well as the phosphorylated form of Smad2 (pSmad2) throughout the adult brain including the neurogenic regions [38]. Further, extensive investigation of the pSmad2 expression in the hippocampal neurogenic niche using co-immunolabeling analysis revealed that proliferating subpopulations of Sox2 positive neural stem cells, DCX-positive neuroblasts and GFAP-positive astrocytes failed to show immunoreactivity for pSmad2, suggesting that they were not under TGF-β stimulation. However, mitotically inactive NSCs, neuroblasts as well as mature neurons exhibit a high-level nuclear expression of pSmad2 in the adult hippocampus [38, 154]. Strikingly, glial cells such as GFAP-positive astrocytes and IBA-1 positive microglia are characterized by the expression of TGF-β [76, 224]. However, these immunological cells of the brain appear to be devoid of pSmad2 [38, 58]. Thus, it suggests that glial cell-specific TGF-β mediates paracrine signaling in the NSC niche rather than autocrine signaling in the adult brain. Further, we observed the direct effect of TGF-β on the regulation of neurogenesis in the brain through intraventricular infusion or a transgenic system expressing TGF-β in the brain under the doxycycline-controlled Ca-Calmodulin kinase promoter [38, 58, 225]. Elevation of TGF-β reduced proliferation of NSCs but promoted the survival of neuroblasts [38]. Besides, in an in vitro study, incubation of neurosphere cultures with TGF-β pushed the cells into the G0 phase of the cell cycle [38, 225]. Subsequently, the above-mentioned facts were confirmed in the brains of transgenic animals that overexpress TGF-β1 under the control of the GFAP promoter in astrocytes [77]. Taken together, it has been hypothesized that TGF-β mediated pSmad2 signaling is responsible for the maintenance of NSCs by inducing cellular quiescence as well as the determination of neural lineage of NSCs and neuroprotection. Moreover, as the expression of TGF-β increases in the aging brain, it might contribute to a decline in neurogenesis [54]. There are some studies indicate that overexpression of TGF-β can induce cell death in NSCs in the hippocampus of the adult brain [226]. Notably, aging brains and many neurocognitive disorders have been characterized by pathologically elevated levels of TGF-β and defects in regenerative plasticity [227]. Hence, it has been conceptualized that impaired neurogenesis in aging and dementia can be attributed to the pathomechanism of aberrant TGF β pathway.

## Regulation of neurogenesis in VaD and the role of TGF-β

In an animal model of VaD, a 2-vessel occlusion (2VO) in experimental rats mimicking chronic cerebral hypoperfusion (CCH) [228, 229], hippocampal neurogenesis is more severely reduced in association with cognitive impairments [230]. Physical exercise rescues the deficits in hippocampal neurogenesis and cognitive functions through the BDNF-CREB pathway in 2VO models [231]. In another study reported by Kwon et al., the inhibition of acetylcholinesterase promotes hippocampal neurogenesis and cognitive function in VaD [232]. Moreover, transplantation of MSCs provided neuroprotection in the hippocampus of 2VO models [233]. Reports on the effects of VaD induced TGF-β levels on adult neurogenesis are limited. As many animal models of AD display a vascular pathology, the regulation of adult neurogenesis by TGF-β can be expected to be similar in VaD as it is described in AD [234, 235]. In corroboration with existing findings on AD and reports generated from experimental models of VaD, it can be presumed that abnormal levels of TGF-β in association with impaired neurogenesis might provide a potential mechanism for vascular abnormalities mediated defects in regenerative plasticity (Fig. 4).

## Role of TGF-β signaling in neuroprotection and neuroregeneration in neurodegenerative disorders with dementia

In many neurocognitive disorders, reactive astrogliosis, microglial activation, neuroinflammation, defects in the turnover of NSCs, reactive neuroblastosis and progressive neurodegeneration of newly generated as well as existing neurons are part of the underlying pathomechanisms of the disease [58, 236]. Among them, regenerative failure resulting from abnormal neurogenesis contributes to cognitive deficits in dementia [223]. Thus, understanding the mechanism of regeneration in the adult brain and its failures has become increasingly important. Chronic high levels of TGF-β exacerbate pathomechanisms responsible for the development of dementia [38, 227, 234]. The expression of TGF-β and its downstream signaling appears to be drastically increased in the brain in response to various acute brain pathologies like ischemic stroke, brain trauma and epilepsy [213, 237, 238]. In addition, the CSF of patients with AD, Huntington’s disease (HD), Parkinson's disease (PD), Amyotrophic lateral sclerosis (ALS), Spinocerebellar ataxia (SCA) and Multiple system atrophy (MSA) shows increased amounts of TGF-β [155, 239, 240]. Notably, increased TGF-β levels are correlated with the occurrence of reactive neurogenesis in post-mortem brain tissue derived from many of these diseases including AD, HD and ALS [241, 242]. Moreover, many neurodegenerative disorders have been characterized by stem cell quiescence and reactive neurogenesis along with elevated levels of TGF-β signaling during the early phases of pathogenesis [58, 154, 234, 235]. HD is an autosomal dominant neurodegenerative condition and is considered as a hereditary form of dementia, for which numerous experimental models are available [58, 243-245]. Among them, almost all transgenic rodent models of HD show elevated levels of TGF-β signaling in association with NSC quiescence and reactive neuroblastosis, impaired neuronal differentiation and degeneration of new neurons [58, 154, 227]. The increased level of pSmads in NSCs blocks the neurogenic process at an early stage in the hippocampus of the R6/2 mouse and tgHD rat models [154]. Besides, the induced expression of TGF-β mediated pSmad2 increases the expression of the mutant form of huntingtin, thereby leading to neurodegeneration [227]. In addition to animal models of HD, increased levels of TGF-β and a reduced level of neurogenesis were reported in genetic models of AD [61, 246, 247]. Moreover, reduced TGF-βR2 expression and defects in the phosphorylation of Smad proteins are reported to be associated with an increased level of amyloidogenesis and hyper-phosphorylation of Tau in the brain of AD subjects [248-250]. Thus, available reports on the regulation of neurogenesis in response to the abnormal levels of TGF-β appears somewhat inconsistent in AD [250-252], but this may relate to the stage of the disease and degree of the pathology.

Several studies have indicated that the induction of TGF-β during pathogenesis exerts a defensive role in preventing neurodegeneration [219]. Reduced expression of TGF-β and deficient Smad signaling in neurons induces neurodegeneration in the terminal stage of the disease [61]. The reduced neurogenesis seen in the brains of some models of AD could provide a possible explanation for the reduction in TGF-β mediated neurodegeneration in cognitive centers of the pathological brain. Recently, reactive neuroblasts in the diseased brain have been proposed to acquire properties of non-neuronal cells such as microglia, and thus these cells might represent a potential source of TGF-β in pathological brains [223, 253]. The robust activation, proliferation and circulation of microglia, reactive astrocytes and neuroblasts found in early pathogenesis tend to be diminished in the late phases of the diseases [227, 254]. Thus, it can be hypothesized that the overall loss of neuroblasts, non-neuronal and neuronal cells in the brain in the late phase of the disease might lead to the reduced level of TGF-β, thereby fostering neurodegeneration and memory loss.

**Figure 5.**
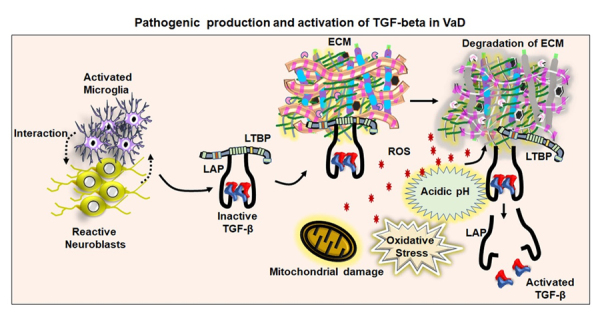
**A proposed mechanism for TGF-β activation in the VaD**. The figure represents the proposed hypothesis that interaction between neuroblasts and activated microglial cells might lead to the production of the inactive form of the TGF-β attached with latent transforming growth factor-β binding protein (LTBP) through latency-associated peptide (LAP). The inactive form of TGF-β has generally been stored in the extracellular matrix (ECM). Vascular damage upon mitochondrial defects, release of free radical and acidification of the local microenvironment might be underlying mechanisms of the pathological activation and release of TGF-β in VaD.

## A proposed mechanism of TGF-β secretion and activation in VaD

While the potential cellular sources of the increased TGF-β levels are identified, the mechanism for the activation of TGF-β remains unknown in neurodegenerating brains. A possible interaction between dying cells and immune cells is proposed to be involved in the production of TGF-β, which in turn can mediate the inflammatory response and oxidative stress through the generation of inflammatory factors and reactive oxygen species [255, 256] (Fig. 5). Overproduction of free radicals causes oxidative damage to molecular and subcellular components of the cell, thereby leading to cell death in many tissues [257]. Oxidative stress plays a central role in inducing neurodegeneration in many dementia cases [258]. However, the effect of free radical-mediated oxidative stress on the activation of TGF-β has not been clearly established in neurocognitive disorders including VaD. While the increased level of free radicals in the vasculature and reduced bioavailability of vasodilators are associated with endothelial dysfunction leading to vascular dementia, free radicals can lead to acidosis in vascular disorders in association with the degradation of ECM [259]. As mentioned in an earlier chapter, the inactive form of TGF-β can be activated by the free-radical mediated increased acidic condition of the tissue [260]. Thus, the increased level of free radicals might be an underlying cause for triggering the activation of TGF-β in VaD. At the same time, in order to release the active form of TGF-β, the ECM needs to be degraded, which may result in the disruption of the cerebrovascular structure and breakdown of BBB. Thus, free radical-induced activation of TGF-β through the generation of acidic microenvironment could be hypothesized to play a central role in the development of VaD. Recently, Unger MS et al. provided evidence for the association of DCX-expressing T cells and microglia in the vicinity of amyloid plaques in the brain of APP-PS1 transgenic mice and human AD subjects [261]. Moreover, it could be shown that DCX-expressing immune cells exhibit indications of phagocytosis in AD brains [261]. Thus, it can be hypothesized that a potential interaction between the DCX-expressing cells and microglia may be a potential source of TGF-β during neuropathogenesis. Eventually, the elevated TGF-β might be an underlying factor for impaired neurogenesis responsible for memory loss in VaD. Taken together, TGF-β signaling is an ideal therapeutic target to intervene in pathological associated vascular defects, neurodegeneration and promote regenerative plasticity in VaD.

**Figure 6.**
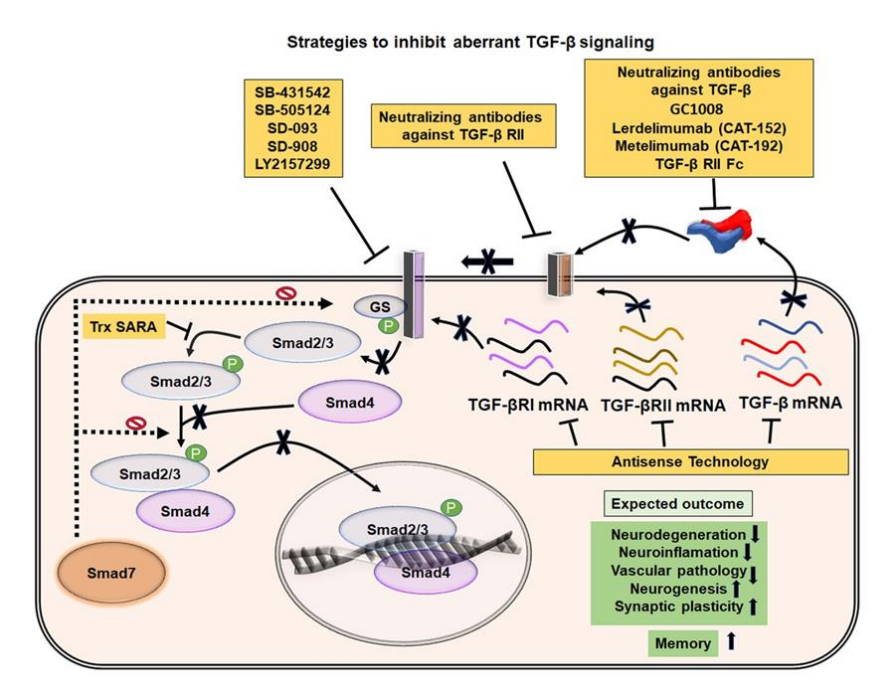
**Possible strategies to inactivate TGF-β signaling and expected outcomes in VaD**. Illustration of possible ways to inhibit aberrant TGF-β signaling pathways. The activities of TGF-β and TGF-β receptors can be neutralized using various recombinant antibodies or their translation can be blocked using specific set of antisense oligonucleotides. Different synthetic molecules that can inhibit the phosphorylation of the TGF-β RII and Smad2/3 are also indicated. The blockade of pathogenic TGF-β signaling has been expected to mitigate the cognitive deficits through inhibition of various neuropathogenic events and promotion of neuroregenerative plasticity in VaD.

## Strategies to modulate TGF-β signaling to mitigate pathogenesis and regenerative potentials in VaD

Town T et al. reported that the inhibition of TGF-β signaling in peripheral immune cells by crossing the CD11c-DNR mice that express the CD11c promoter-driven dominant-negative TGF-β receptor type 2 with Tg2576 mice overexpressing mAPP reduced the brain parenchymal and cerebrovascular β-amyloid deposits and improved the working memory [262]. Therefore, inhibition of overactive TGF-β signaling has been proposed to attenuate neurovascular and neuro-regenerative pathologies in VaD.

TGF-β can be modulated through several distinct approaches (summarized in Figure 6). The main strategies for inhibition of TGF-β include neutralizing antibodies and recombinant fragments that block TGF-β, implementation of compounds and antisense oligonucleotides that interfere with the binding of the ligand to TGF-β receptors, and small molecules that block intracellular phosphorylation of TGF-β receptor 1 and Smad elements. Lerdelimumab (CAT-152) and metelimumab (CAT-192) are recombinant human IgGs generated by phage display technology that block TGF-β1 and TGF-β2 respectively [263]. Recombinant fusion proteins containing the ectodomains of type 2 and type 3 receptors, the targeted pan-TGF-β blocker (TTB) and soluble TGF-β II/Fc have also been used to prevent ligand binding to TGF-β receptors [264]. The synthetic agents such as SB-431542, SB-505124, SD-093, SD-908 and LY2157299 were identified to block the catalytic activity of TGF-βR1 [265]. Moreover, the antisense oligonucleotides generated against the expression of TGF-β or the TGF-β receptors can also be a promising approach to block TGF-β signaling to eventually treat VaD at an early stage of the pathogenesis. However, the safety and efficacy of these agents need to be verified in preclinical and clinical trials. Moreover, inhibition of TGF-β activity may elicit the risk for the anomalies in cell cycle regulation, cytoprotection, immunity and metabolism. Thus, unknown adverse effects of the inhibition of TGF-β related to its normal biological functions and the toxic dosages of the TGF-β agonist may not completely be excluded. Moreover, the overlapping pathophysiology between the VaD and AD needs to be dissected out, for which, the development of a valid experimental model to mimic solely the VaD that reflects the human situation needs to be accomplished. However, further studies are needed to appraise the discussed information and proposed ideas of this article for the most relevant clinical information.

## Conclusion

There is ample scientific evidence that strongly supports that aberrant TGF-β signaling is the main underlying mechanism for the pathogenesis of VaD. Increasing evidence has led to TGF-β receiving great scientific attention in recent years because it may play a critical role in the development and progression of neurovascular pathologies and neurocognitive disorders including dementia. Systemic and cerebral alteration of TGF-β in association with the morphological changes of the cerebrovascular system and progressive cognitive disruption may serve as a potential biomarker for VaD. In particular, the multiple aspects of the pathology of VaD where TGF-β might be centrally involved makes the inhibition of TGF-β signaling an attractive and multimodal approach to address the various aspects of VaD. Therefore, the inhibition of aberrant TGF-β might be an ultimate therapeutic strategy for the treatment of VaD subjects.
